# Research Hotspots and Trends of RNA Research in Diabetic Retinopathy: Insights From Bibliometric Analysis

**DOI:** 10.2174/0118715303358069241213151650

**Published:** 2025-01-03

**Authors:** Dan Yin, Xianke Luo, Maoying Wei, Yiting Tang, Yijia Jiang, Churan Wang, Aijing Li, Jingyi Guo, Yanbing Gong

**Affiliations:** 1 Department of Endocrinology, Dongzhimen Hospital, Beijing University of Chinese Medicine, Beijing, China;; 2 Department of Ophthalmology, Chengdu First People’s Hospital, Chengdu, China;; 3 Teachers' Work Department, Beijing University of Chinese Medicine, Beijing, China

**Keywords:** Diabetic retinopathy, RNA, non-coding RNA, bibliometrics, VOSviewer, CiteSpace

## Abstract

**Background:**

Research on RNA in diabetic retinopathy (DR) has received increasing attention in recent years. However, there is a lack of comprehensive and systematic research on the current status and future direction of RNA research in DR. Therefore, this study used bibliometric analysis to summarize the global trends and current status of DR RNA research to date.

**Methods:**

We searched the Web of Science for studies related to DR RNA research before July 2024 and then used CiteSpace and VOSviewer to generate knowledge visualization maps.

**Results:**

A total of 1,103 articles related to RNA research in DR were retrieved. China has the most articles, followed by the USA and Japan. Wayne State University, Nanjing Medical University, and Shanghai Jiao Tong University were the three most productive institutions. Investigative Ophthalmology & Visual Science was the most popular journal in this field. Kowluru, Renu A from Wayne State University published the most number of articles. Keyword analysis showed that the research hotspot in this field is the role of miRNAs in apoptosis and neovascularization in DR. Non-coding RNAs and extracellular vesicles are future research trends.

**Conclusion:**

The results of this bibliometric study provide information on the current status and trends in the field of RNA research in DR. RNA research could lead to new diagnostic methods and therapeutic strategies for DR. Our findings can help researchers understand the current status of RNA research in DR and identify new directions for further research.

## 1 INTRODUCTION

Diabetes mellitus is an endocrinal disorder [1, 2], and diabetic retinopathy DR is a severe complication of diabetes mellitus and a leading cause of adult blindness worldwide [3]. Epidemiological data from 2021 indicate that more than 500 million people had diabetes [4], with about one-third of them at risk of developing DR [5]. Estimates suggest that by 2045, up to 160 million people could have DR. Effective glycemic control, blood pressure optimization, and lipid management can help reduce or slow the progression of DR [6]. Early detection and treatment of advanced DR stages, including pan-retinal photocoagulation and intravitreal anti-VEGF injections, can lower the risk of vision loss [7]. Nonetheless, around 50% of patients may not benefit significantly from anti-VEGF injections, and laser photocoagulation can be detrimental and not always effective [8].

With advances in human genomics, gene therapy has emerged as a promising treatment approach for various diseases [9]. Previous studies have found that 131 circular RNAs (circRNAs) [10], 8 microRNAs (miRNAs) [11], and 427 long non-coding RNAs (lncRNAs) [12] are differentially expressed during the pathogenesis of DR compared to normal tissues, suggesting that some of the RNA molecules are closely associated with the onset and progression of DR. In particular, miRNAs have been a hot research topic in the last 20 years, and they play an important role in human metabolism, cell division, cell proliferation, cell differentiation, and apoptosis *etc* [13]. Some specific miRNAs, such as miR-21 and miR-29b, play a role in the regulation of key pathways of DR, including neovascularization, fibrosis, and inflammation [14]. In addition, Zhu *et al.* observed an upregulation of miR-20b-5p in both diabetic rats and human retinal microvascular endothelial cells (HRMECs) [15]. The inhibition of miR-20b-5p resulted in elevated levels of tight junction proteins, including zonula occludens 1 (ZO-1), occludin, and claudin-5. This increase in tight junction protein expression is associated with enhanced blood-retinal barrier permeability, microvascular leakage, and retinal damage. In diabetic mice, silencing circHIPK3 led to a reduction in retinal acellular capillary number and vascular leakage, while overexpression of circHIPK3 reversed these effects [16]. This knowledge emphasizes the therapeutic potential of targeting miRNAs to reduce retinal damage and vascular leakage.

In recent years, academic literature in the area of RNA research in DR has increased dramatically. Given the proliferation of publications, traditional analysis methods are no longer sufficient to fully assess the evolution of this area of research. Bibliometric analysis is a widely used method to systematically review medical research. By quantitatively analyzing literature, this method not only reveals trends within the research field but also provides a structured evaluation of advancements across various disciplines, countries, institutions, and authors. Consequently, it is crucial to outline the features and future directions of a discipline. Previous studies have been bibliometrically analyzed for DR, such as studies on stem cell therapy, artificial intelligence in DR [17, 18]. However, it lacks bibliometric study of RNA in DR. This study aimed to perform a bibliometric analysis of RNA research in DR within the Web of Science Core Collection (WoSCC) using tools such as CiteSpace and VOSviewer, offering new insights for researchers in this area.

## 2 MATERIALS AND METHODS

### 2.1 Data Source and Search Strategy

We performed a comprehensive search and analysis using the Web of Science Core Collection (WoSCC) database, focusing on English-language publications up to 16 July 2024. WoSCC was chosen due to its inclusion of numerous esteemed and impactful journals preferred by scholars [19, 20]. The search strategy was as follows: (TI = “Ribonucleic Acid” OR “Transfer RNA*” OR tRNA* OR RNA* OR rRNA* OR “Messenger RNA*” OR mRNA*) OR (TS = (microRNA* OR miRNA* OR “long non-coding RNA*” OR lncRNA* OR “short non-coding RNA*” OR “small non-coding RNA*” OR sncRNA* OR “small nuclear RNA*” OR snRNA* OR “small nucleolar RNA*” OR snoRNA* OR “Non-coding RNA*” OR ncRNA* OR “small RNA*” OR sRNA* OR “Small interfering RNA*” OR “small interference RNA*” OR siRNA* OR “PIWI-interacting RNA*” OR piRNA* OR “Circular RNA*” OR circRNA*)) AND (TS = (“Diabetic Retinopathy”)). The literature type was restricted to articles and reviews, and the language was English. All relevant literature concerning RNA in DR was exported in TXT format as “Full Records and References.” The flowchart of article inclusion is shown in Fig. (**1**).

### 2.2 Data Analysis

To improve the presentation of our research findings, we meticulously chose appropriate software to analyze the different aspects of our study. In this investigation, we utilized VOSviewer software (version 1.6.18) to facilitate the visualization of countries, institutions, authors, journals, references, and keywords co-occurrence networks. In these visualizations, the node size indicates the frequency of appearance or citation, while the width of the connecting lines reflects collaboration strength. The threshold for the country co-authorship network was set to 1. The keyword co-occurrence network displayed keywords with a frequency above 20, and the top five frequencies were shown for each of the remaining categories. The default settings were used for the other software parameters. Additionally, we used CiteSpace software (version 6.3 R1) to generate a journal double map and keywords bursts map.

## 3 RESULTS

### 3.1 Annual Publications

The quantity of published papers can, to some degree, indicate shifts in researchers' focus on a specific field. We gathered a total of 1,103 publications from the WoSCC database, which includes 942 articles and 161 reviews. A bar chart was provided to show the trend in the number of RNA-related publications in DR from 1992 to 2024. Fig. (**2**) demonstrates that the annual publication count has generally increased, suggesting a growing interest in RNA research in DR field.

### 3.2 Distribution of Countries/Regions

Between 1992 and 2024, 52 countries or regions from six continents have contributed to RNA research in DR. Table **1** illustrates the top ten most productive nations, with China leading as the most prolific, having 606 publications and 14,008 citations, accounting for 54.94% of the total publications. The USA (252 publications, 11,454 citations) followed at 22.85%, while Japan (45 publications, 1,622 citations) ranked third with 4.08%. Notably, China and the USA accounted for 77.79% of the total number of publications, underscoring a high level of interest in RNA research in DR.

We used VOSviewer to assess collaborative authorship across countries and regions. The USA not only held the second position in total publications but also demonstrated the strongest total link strength and a notably high average citation rate (Table **1**), reflecting its advanced research in RNA studies related to DR. Through co-authorship analysis, VOSviewer grouped countries/regions into five distinct clusters. As depicted in Fig. (**3**), the red cluster, centered around the USA, China, and Japan, includes 14 countries, with China and the USA showing particularly strong collaboration. The green cluster features countries like Italy, Germany, Spain, and France. The blue cluster is composed of Australia, England, Denmark, and other countries. India collaborates closely with Canada, Iran, Iraq, Thailand, and other nations (yellow). The purple cluster contains Argentina, Ecuador, and Israel. The analysis of annual publications and countries distribution showed that many countries, including the USA, China and India, were increasingly interested in the investigation of RNA in DR. Meanwhile, the increasing international collaboration in this field confirms the importance of RNA research in the development of diagnostic and therapeutic options for DR.

### 3.3 Institution Analysis

We visualized the collaboration between research institutions with a minimum of five documents. The results, drawn from 1,151 institutions, identified 82 institutions that meet this threshold. As illustrated in Fig. (**4**), there was substantial collaboration among these institutions, which contributed significantly to the advancement of the field.

Table **2** shows the most productive research institutions. Among them, nine institutions are based in China, while one institution is from the United States (Wayne State University). Wayne State University (n = 57) leads in publication output, with Nanjing Medical University (n = 50) and Shanghai Jiao Tong University (n = 39) following. Additionally, Wayne State University has the highest average citation count (54.23), followed by Nanjing Medical University (55.30) and Fudan University (53.30). In terms of total link strength, Nanjing Medical University leads the top ten institutions with a score of 33, followed by Shanghai Jiao Tong University (n = 28) and Fudan University (n = 27). This indicates that these institutions maintain particularly strong connections with others in RNA research related to DR.

### 3.4 Author Analysis

This study investigated 9,572 authors and 44,606 co-cited authors. Table **3** shows that Kowluru, Renu A is the most productive author, with 33 published articles. The following are Steinle, Jena J (17 articles), and Chakrabarti, Subrata (15 articles). Additionally, Kowluru, and Renu A had the highest total citations (1,947 citations), while Yan and Biao had the highest average citations and total link strength. Their research significantly impacted the field of RNA in DR. Fig. (**5**) illustrates a co-authorship map featuring publications with more than five papers. Notably, Kowluru, Renu A collaborates closely with Mishra, Manish, and Mohammad, Ghulam. Yan and Biao collaborated closely with Jiang, Qin; Liu, Chang; Li, Xiu-Miao; Yao, Jin; and Shan, Kun. Chakrabarti and Subrata collaborated closely with Feng, and Biao, while Steinle and Jena J worked closely with Jiang, Youde and Liu, Li. Yao, Yong collaborated with Li, Yan and Shao, Jun, and Roy, Sayon worked closely with Kim, Dongjoon. Furthermore, authors who are cited by two or more other authors in one or more publications are referred to as co-cited authors. The top ten co-cited authors are listed in Table **3**. Kowluru, Renu A (493 citations), Antonetti, Da (145 citations), and Cheung, N (143 citations) are the leading co-cited authors, having made crucial contributions to the knowledge base of RNA research in DR.

### 3.5 Journal Analysis

Table **4** lists the top ten most productive journals. Investigative Ophthalmology & Visual Science (n = 57), Experimental Eye Research (n = 37), and Scientific Reports (n = 25) were the top three academic journals that published articles on RNA research in DR out of 367 academic journals. In addition, Investigative Ophthalmology & Visual Science recorded the highest number of total citations, while Diabetes achieved the highest average citations. The journal impact factor (IF) serves as a key metric for gauging the influence of academic journals, including assessing their reputation, research quality, and the significance of their research outcomes [21]. Diabetes had the highest impact factor (IF 2023 = 6.2), followed by Investigative Ophthalmology & Visual Science (IF 2023 = 5) and International Journal of Molecular Sciences (IF 2023 = 4.9). According to Journal Citation Reports (JCR), six of the top ten most productive journals fall within the Q1 region.

The superposition of double maps illustrates the distribution of topics across journals. The map labels indicate the range of research fields covered by journals. On the left side of the map are citing journals, while cited journals are positioned on the right. Lines connecting journals represent citation relationships, showing that research topics in cited journals are frequently referenced by those in citing journals. The thickness of these lines reflects the frequency of citations between topics. A central yellow citation pathway was found only among molecular/biology/ genetics journals, indicating that they were primarily cited by molecular/biology/immunology journals Fig. (**6**).

### 3.6 Reference Analysis

Table **5** provides a list of the top ten co-cited references in the field of RNA research in DR, including five review articles. Martinez *et al.* searched for studies published before 2019 on serum/plasma miRNAs in patients with early or advanced DR and discussed the potential of miRNAs as diagnostic and prognostic biomarkers for DR [22]. Gong and Mastropasqua *et al.* described the potential role of miRNAs and long noncoding RNAs in diabetes and evaluated their implications in DR [23, 24]. Wang and Rübsam *et al.* reviewed the understanding and insights into the pathophysiology of DR in 2018 [25, 26]. In addition, several RNAs that have been studied in the top ten cited papers are listed in Table **6**, and these RNAs have received relatively more attention from researchers.

### 3.7 Keywords Co-Occurrence Networks

High-frequency keywords highlight key research areas within the field. Table **7** presents the ten most frequently occurring keywords in DR RNA research. Furthermore, we utilized CiteSpace software to create a Timeline Viewer, with parameters set to a period of 1992 to 2024, one year per slice, focusing on keywords as the node type, selection criteria (top N=50), and without any pruning. The top ten clusters Fig. (**7**), in descending order, include “diabetic retinopathy,” “growth factor,” “angiogenesis,” “choroidal neovascularization,” “endoplasmic reticulum stress,” “retinal endothelial cells,” “transcriptional regulation,” “small interfering RNA,” “atherosclerosis,” and “Müller cells.” Of these, Research interest in Cluster #0 (“diabetic retinopathy”) and Cluster #3 (“choroidal neovascularization”) continued into 2024, while the other clusters saw a decline in research activity by 2019.

Fig. (**8**) illustrates the top ten keywords with the most significant citation bursts, where the red line represents times of high occurrence frequency. By analyzing these keyword bursts, we can predict future research frontiers. “Biomarkers,” “long noncoding RNA,” “circular RNA,” “extracellular vesicles,” and “non-coding RNA” represent recent frontiers in RNA research related to DR.

## 4 DISCUSSION

### 4.1 Publication Trends and Cooperation

Bibliometric methods were utilized to analyze the research status of RNA in DR from 1992 to 2024. Owing to global interest in DR and the growing understanding of the pathophysiology of RNA in DR, research productivity in this area has shown a gradual upward trend over the past 30 years. With approximately 141 million people living with diabetes in China, accounting for one-third of the world’s total number of diabetic patients, China has demonstrated a significant interest in research in this area. Among the top ten organizations by publication count, nine were from China and one was from the United States, reflecting the overall publication distribution by country. Wayne State University leads with the highest number of publications, while Nanjing Medical University ranks second and shows the strongest connections with other institutions. The close relationships between countries and institutions foster a supportive environment for academic collaboration and advance RNA research in DR.

Among the top ten authors, Professor Kowluru, Renu A stands out as the most prolific with 33 published articles, followed by Steinle, Jena J with 17 and Chakrabarti, Subrata with 15. This highlights the strong and ongoing dedication of these three researchers to RNA research in DR. The key research interests of Kowluru, Renu A include the epigenetics of DR [27], retinal mitochondria [28], and matrix metalloproteinase-9 [29]. Especially in recent years, Kowluru, Renu A has conducted in-depth research on the regulation of mitochondrial stability in DR by long noncoding RNAs and has made outstanding contributions as an expert in this field [30]. Steinle, Jena J focused on the protective effects of miR-146a and miR-15a/16 on the retinal vascular endothelium [31, 32], contributing to the study of miRNAs in DR. Chakrabarti, Subrata investigated the role of miRNAs and lncRNAs in regulating endothelial-mesenchymal transition in DR. He has also published several reviews on the relationship between RNA and DR, summarizing some of the research results in this field.

Fig. (**6**) presents a journal double map representing thematic distribution, in which only a central orange citation path was observed. Research published in molecular/biology/genetics journals is predominantly cited by research published in molecular/biology/immunology journals, indicating that research associated with DR RNA is currently mainly focused on basic research, whereas translational medicine research remains limited.

### 4.2 Research Basics and Hot Spots

#### 4.2.1 Research Basics and Hot Spots Traced from Cited References

Cited references form the foundational knowledge within a research domain. In this study, five out of the top ten most cited references are review articles. Rodolfo Mastropasqua [24], Qiaoyun Gong [23], and Bridget Martinez [22] provided summaries of previous research on the use of miRNAs and long noncoding RNAs (lncRNAs) as diagnostic or prognostic biomarkers for DR and their potential as therapeutic targets. These reviews will assist researchers in gaining a deeper understanding of the molecular mechanisms through which miRNAs and lncRNAs exert their effects in DR, and they will guide future research. Additionally, Table **6** lists several RNAs that were examined in the top ten cited references. Pathological angiogenesis plays a significant role in various diseases, including ocular disorders, cancers, and atherosclerosis. This condition is typically driven by abnormal biological processes such as cell proliferation, motility, immune responses, or inflammation. LncRNAs have been identified as crucial regulators of these processes. However, the involvement of lncRNAs in microvascular dysfunction induced by diabetes mellitus remains largely unexplored. Yan *et al.* (2014) aimed to investigate whether lncRNA-myocardial infarction–associated transcript (MIAT) is implicated in diabetes mellitus–induced microvascular dysfunction [33]. Their study revealed that lncRNA-MIAT expression was elevated in the retinas of diabetic subjects and in endothelial cells cultured in high glucose conditions. Additionally, the knockdown of MIAT significantly improved retinal microvascular dysfunction caused by diabetes *in vivo* and suppressed endothelial cell proliferation, migration, and tube formation *in vitro*. It has been proposed that MIAT acts as a competitive endogenous RNA, forming a feedback loop with vascular endothelial growth factor and miR-150-5p to modulate endothelial cell function. In 2017, Chen *et al.* examined the role of miR-21 in diabetes-induced retinal inflammation and apoptosis [34]. Their findings indicated that miR-21 contributes to DR pathology by regulating PPARα levels in the diabetic retina, suggesting that miR-21 could be a potential therapeutic target for DR. In 2018, Biswas *et al.* studied a lncRNA notorious in cancer, MALAT1, and found that it also plays an important role in DR inflammation and epigenetic regulation [35]. MALAT1 can influence the expression of inflammatory transcripts through its combination with epigenetic mediators, such as histones and DNA methyltransferases. The results of this study are informative for the future establishment and development of new lncRNA models for DR. In 2019, Anu A Thomas and colleagues identified a new function of lncRNA H19 in modulating endothelial–mesenchymal transition triggered by high glucose levels. H19 accomplishes this by inhibiting TGF-β1 and its signaling pathways through repression of the MAPK–ERK1/2 signaling pathway. Nevertheless, other mechanisms potentially influencing these pathways warrant further investigation [36]. This study suggests the potential of RNA therapy to address glucose-mediated changes in cellular phenotypes. Also in 2019, Ke Zhu and team observed that circDNMT3B expression was reduced in both DR patients and diabetic rat retinal endothelial cells. CircDNMT3B influences the proliferation, migration, and tube formation of HRMECs in diabetic conditions by targeting miR-20b-5p and its downstream regulator BMP and activin membrane-bound inhibitor (BAMBI) [15]. CircDNMT3B overexpression reduced the number of capillaries in diabetic retinal cells and attenuated visual impairment.

#### 4.2.2 Exploring Research Hotspots Through Keyword Co-Occurrence

Keywords can effectively reveal the distribution of research hotspots in RNA studies related to DR. Excluding terms like DR, activation, retinopathy, and mechanisms, Table **6** consists of the following keywords: endothelial growth factor, endothelial cells, angiogenesis, apoptosis, microRNAs, and VEGF. Combined with the Timeline Viewer, it can be concluded that microRNAs are the most studied RNA type in the field of DR research, with retinal neovascularization, endothelial cells and related growth factors, and apoptosis identified as research priorities. In conjunction with the Timeline Viewer, the research hotspots in the field of RNA research in DR are summarized as follows:

##### 4.2.2.1 MicroRNAs and Cell Apoptosis

MiRNAs are a class of endogenous, single-stranded, small non-coding RNAs (ncRNAs), ranging from 21 to 25 nucleotides, that regulate target gene expression post-transcriptionally by binding to their 3′-untranslated region (3′-UTR) [37, 38]. They are involved in various cellular processes, including differentiation, cell growth, apoptosis, and inflammation [39]. DR progression is notably marked by the loss of retinal cells, such as neural retinal cells, pericytes, and endothelial cells. Recent studies have shown that miRNAs play a crucial role in DR development by modulating apoptotic pathways. During the development of DR, failure to intervene promptly in blood-retinal barrier damage can lead to vision loss in patients and blood-retinal barrier damage is closely related to pericytes, endothelial cells, and RPE cell apoptosis triggered by inflammatory responses. In this context, Wan *et al.* investigated the protective role of miR-200a in DR progression. They found decreased levels of miR-200a and increased levels of LIM domain protein 1 (PDLIM1) in both *in vivo* and *in vitro* DR models. miR-200a was shown to mitigate apoptotic conditions, improve cell viability, and significantly reduce cellular migration in high-glucose-treated HRMECs by targeting PDLIM1. Thus, miR-200a emerges as a promising therapeutic target for modulating PDLIM1 expression in DR [40]. In DR rat and ARPE-19 cell models, miR-320b regulates high glucose-induced apoptosis and inflammation by mediating the expression of TIMP3. The addition of a miR-320b inhibitor or overexpression of Circ_NNT results in an increase in TIMP3 levels, thereby reducing apoptosis and inflammation [41]. Similarly, inflammatory responses and cellular apoptosis were inhibited after the injection of human umbilical cord mesenchymal stem cells extracellular vesicles (hUCMSCs- EVs) in DR rats and HRMECs. Xu *et al.* carried out differential analysis of miRNAs in hUCMSCs-EVs and found that 69 miRNAs were differentially expressed, and empirically verified that the difference in expression of miR-18b was significant, suggesting that miR-18b could inhibit inflammatory responses by regulating mitogen-activated protein kinase 1 expression to inhibit the phosphorylation of NF-κBp65, exerting anti-inflammatory and anti-apoptotic effects, thereby alleviating the development of DR [42].

##### 4.2.2.2 MicroRNAs and Neovascularization

The imbalance between vascular endothelial growth factor (VEGF) and anti-VEGF factors is crucial in the progression from non-proliferative DR (NPDR) to proliferative DR (PDR). VEGF, a key angiogenic factor, plays a significant role in mediating ischemia-induced neovascularization in the retina [43]. Compared with NPDR patients, PDR patients had lower serum levels of miR-15b and higher levels of VEGF, and in the HRMECs model of DR, increased miR-15b inhibited neovascularization by a mechanism in which miR-15b negatively regulates the expression of the downstream target gene VEGF [44]. Moreover, miR-21 has been linked to retinal neovascularization through its regulation of peroxisome proliferator-activated receptor-α (PPARα). In mouse models, miR-21 overexpression results in reduced levels of PPARα, which has been implicated in promoting retinal neovascularization. Knockdown of miR-21 prevents PPARa from being reduced, thereby reducing retinal neovascularization and inflammation [34].

Kara *et al.* reported decreased levels of miR-200b in the retinas of diabetic patients, alongside increased mRNA and protein levels of VEGF, a target of miR-200b. miR-200b is found in neuronal, glial, and vascular components of the retina. Transfecting endothelial cells or administering miR-200b mimics intravitreally counteracts the diabetes-induced rise in VEGF mRNA and protein levels, as well as glucose-induced increases in permeability and angiogenesis [45]. Wang *et al.* observed that miR-30b was up-regulated in the retinal tissues of PDR mice. In a high glucose-induced retinal microvascular endothelial cells (RMECs) model, reducing miR-30b expression led to decreased VEGF levels and inhibited neovascularization by increasing SIRT1 expression [46]. Anti-VEGF therapy is known to be widely used in the clinical management of DR, but its efficacy remains limited and adverse side effects may occur [47, 48]. Numerous previous studies have confirmed that miRNA has a positive role in regulating VEGF expression and inhibiting retinal neovascularization. These miRNA-based therapies are consistent with the objectives of precision medicine, offering patients with DR more personalized treatment options.

#### 4.2.3 Exploring Research Frontiers Through Keyword Burst Analysis

Keyword burst analysis identified that “biomarker,” “long non-coding rna,” “circular rna,” “extracellular vesicles,” and “non-coding rna” represent the latest frontiers of RNA research in DR. In recent years, ncRNAs, including circRNAs and lncRNAs, have been proposed to regulate gene expression and affect various biological processes in DR [49].

CircRNAs are a distinctive class of endogenous ncRNAs, widely expressed in mammalian serum and noted for their higher abundance, specificity, and organization compared to other RNA types [50]. Recent research indicates that circRNAs can function as sponges for miRNAs and proteins, and also influence gene expression, epigenetic modifications, peptide translation, and pseudogene formation [51]. Identified and characterized in DR patients, circRNAs are now considered novel therapeutic targets for the disease [52, 53]. Studies reveal that certain circRNAs are crucial in DR development, impacting retinal endothelial cell proliferation, migration, and endothelial angiogenesis [52]. Besides circRNAs, lncRNAs are another frontier in the field of DR RNA research. LncRNAs are ncRNAs of more than 200 nucleotides [54] that play important roles in many processes, including viral infections [55], metabolism [56], cell cycle regulation, epigenetic regulation [57], and regulation of cell differentiation [58]. There is growing evidence that lncRNAs regulate miRNAs during DR progression and modulate the expression of related genes in transcriptional or epigenetic mechanisms, thereby influencing DR progression [23].

The study of miRNA biomarkers for DR has also received close attention in recent years. miRNAs can be stably present in serum and other body fluids, and due to their endogenous, stable and noninvasive biopsy characteristics, they have been used as biomarkers for many diseases, such as diabetes mellitus, diabetic nephropathy, DR, ophthalmic diseases, cardiovascular diseases and various cancers [59-65]. For example, Qin *et al.* found that serum miR-126 could be used as a noninvasive biomarker for screening retinal endothelial damage and early diagnosis of non-proliferative DR [66]. Yang *et al.* used RNA sequencing to analyze the expression profiles of serum-derived exosomal miRNAs and found that miRNA-3976 has the potential to serve as a biomarker for DR [67]. The study of early non-invasive biomarkers of mirna has a positive effect on promoting early intervention and improving patient prognosis.

In addition, research interest in extracellular vesicles (EVs) in this area has increased rapidly in recent years. The continuously accumulating evidence strongly supports the view that EVs, as important mediators of intercellular and tissue-to-tissue communication, are key participants in the pathogenesis and progression of DR [68]. For instance, patients with PDR exhibit higher concentrations of plasma EVs compared to those with NPDR or non-diabetic retinopathy [69]. In addition, through exosome engineering, the expression of relevant ncRNAs in exosomes can be controlled to regulate DR development [70]. Previous studies have shown that non-coding RNA and EVs are likely to play an important role in the development of novel therapeutic approaches such as regenerative medicine and gene therapy. In recent years, gene therapy has gained momentum as a viable treatment for a range of ophthalmic diseases, including both traditional inherited retinal disorders [71] and multifactorial retinal disorders such as Age-related Macular Degeneration [72], uveitis [73] and DR [74]. In addition, it is notable that Professors Victor Ambros and Gary Ruvkun have been awarded the 2024 Nobel Prize in Physiology or Medicine for the discovery of microRNA and its role in post-transcriptional gene regulation, which will greatly encourage researchers to be active in miRNA research. Therefore, future miRNA research is most likely to be a critical area for further investigation in DR.

## 5 LIMITATIONS

Similar to previous bibliometric studies, our study has some limitations [75]. First, to ensure the accuracy of the analysis, we chose the WOS core database for our study, and the results were limited to English-language articles, which may have resulted in missing data from the literature. Second, while CiteSpace and VOSviewer are valuable tools, they cannot fully substitute systematic searches. Finally, bibliometrics cannot assess individual literature. Newer publications may have fewer citations than older ones. These limitations may slightly influence the results of the bibliometric analysis but are unlikely to significantly affect the overall study.

## CONCLUSION

This study provides a bibliometric analysis of RNA research in DR concerning year, country, institution, authors, journals, references, and keywords. The volume of literature has been trending upward every year, demonstrating continued interest in the field. By visually analyzing incoming countries, institutions, authors, and journals, it is possible to present their scholarly contributions without subjectivity while providing potential collaboration opportunities for future research. An overview of the literature and keywords in this field revealed research priorities in the field of RNA research in DR, mainly the role of miRNAs in the regulation of apoptosis and retinal neovascularization in DR cells. In addition, ncRNAs in DR, including circRNAs and lncRNAs, as well as extracellular vesicles, are research frontiers in this field. Peer scholars can refer to this analysis to gain insights into the direction of research in this area.

## Figures and Tables

**Fig. (1):**
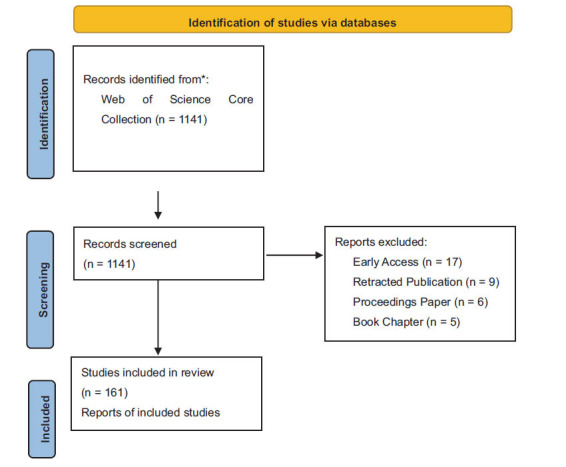
PRISMA Flowchart of the literature search and selection process.

**Fig. (2):**
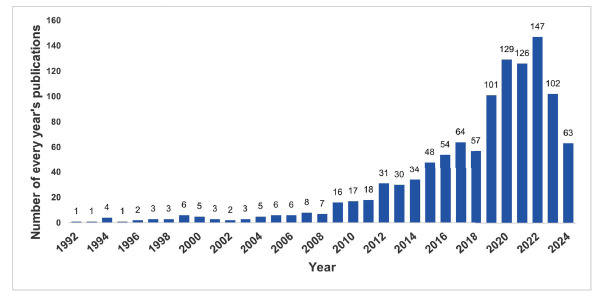
Annual number of publications worldwide.

**Fig. (3):**
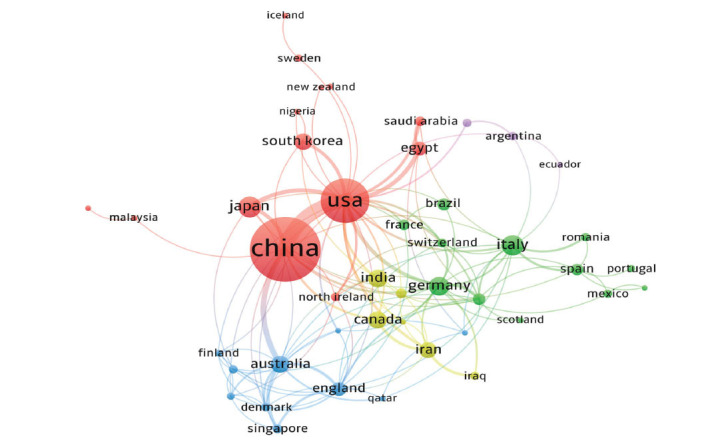
Visualization map of country/region collaboration. Nodes (circles) in the map represent countries/regions. The larger the circle, the more documents are produced by that country/region. A link between two nodes indicates that the two countries/regions have a cooperative relationship, the thicker the line the closer the cooperation. Different colors represent different clusters, and nodes within clusters are relatively more connected.

**Fig. (4):**
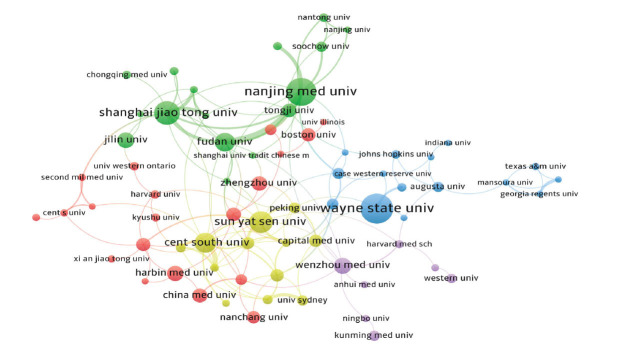
Visualization map of institutional collaboration. Nodes (circles) in the map represent institutes. The larger the circle, the more documents are produced by that institute. A link between two nodes indicates that the two institutes have a cooperative relationship, the thicker the line the closer the cooperation. Different colors represent different clusters, and nodes within clusters are relatively more connected.

**Fig. (5):**
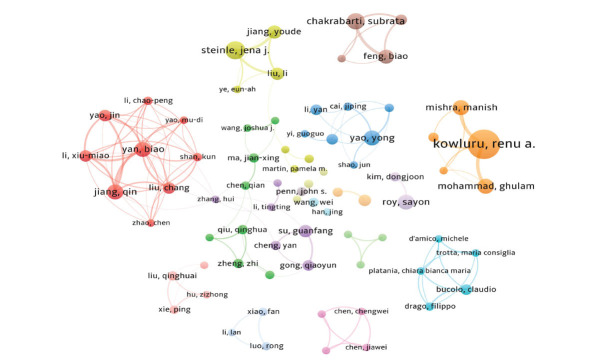
Visualization map of author collaboration. Nodes (circles) in the map represent authors. The larger the circle, the more documents are produced by that institute. A link between two nodes indicates that the two authors have a cooperative relationship, the thicker the line the closer the cooperation. Different colors represent different clusters, and nodes within clusters are relatively more connected.

**Fig. (6):**
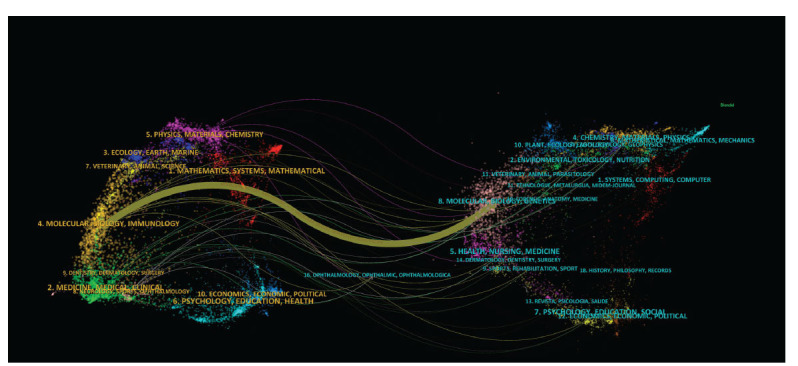
Dual-map overlay of journals. The citing journals are on the left, the cited journals are on the right, and the colored path represents the citation relationship.

**Fig. (7):**
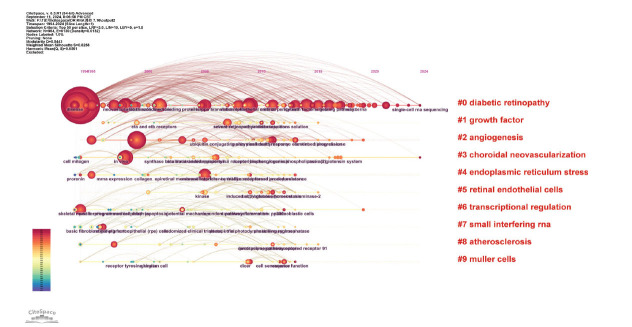
Timeline view for keywords.

**Fig. (8):**
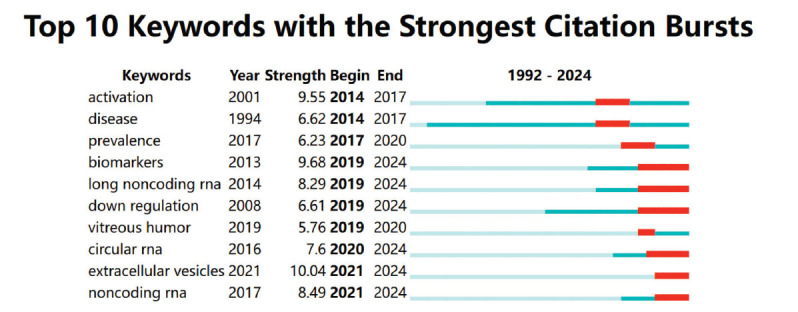
Top 10 keywords with the strongest citation bursts.

**Table 1:** Top 10 most productive countries.

**Rank**	**Countries **	**Documents**	**Total Citations**	**Average Citations**	**Total Link Strength**
1	China	606 (54.94%)	14008	23.12	70
2	USA	252 (22.85%)	11454	45.45	107
3	Japan	45 (4.08%)	1622	36.04	17
4	Italy	42 (3.81%)	1274	30.33	22
5	Germany	34 (3.08%)	1200	35.29	31
6	India	29 (2.63%)	748	25.79	9
7	Australia	27 (2.45%)	732	27.11	33
8	Canada	27 (2.45%)	1343	49.74	12
9	South Korea	25 (2.27%)	686	27.44	8
10	Iran	23 (2.09%)	277	12.04	18

**Table 2:** Top 10 most productive research institutions.

**Rank**	**Institutions**	**Countries**	**Documents**	**Total Citations**	**Average Citations**	**Total Link Strength**
1	Wayne State University	The United States	57	3091	54.23	7
2	Nanjing Medical University	China	50	2765	55.30	33
3	Shanghai Jiao Tong University	China	39	1059	27.15	28
4	Sun Yat-sen University	China	33	830	25.15	20
5	Central South University	China	29	527	18.17	26
6	Fudan University	China	27	1439	53.30	27
7	Wenzhou Medical University	China	21	487	23.19	9
8	Jilin University	China	20	503	25.15	3
9	Harbin Medical University	China	19	442	23.26	4
10	Zhengzhou University	China	18	317	17.61	3

**Table 3:** Top 10 most productive authors and co-cited authors.

**Rank**	**Authors**	**Countries**	**Documents**	**Total Citations**	**Average Citations**	**Total Link Strength**	**Co-Cited Authors**	**Total Citations**
1	Kowluru, Renu A	Wayne State University	33	1947	59.00	38	Kowluru, Renu A	493
2	Steinle, Jena J.	Wayne State University	17	470	27.65	25	Antonetti, Da	145
3	Chakrabarti, Subrata	Western University	15	1058	70.53	25	Cheung, N	143
4	Jiang, Qin	Nanjing Medical University	13	1335	102.69	48	Hammes, Hp	134
5	Mishra, Manish	Wayne State University	13	925	71.15	19	Aiello, Lp	127
6	Mohammad, Ghulam	Wayne State University	13	453	34.85	16	Bartel, Dp	119
7	Roy, Sayon	Boston University	13	498	38.31	7	Gong, Qy	119
8	Yan, Biao	Fudan University	13	1710	131.54	52	Zhang, Y	118
9	Yao, Yong	Nanjing Medical University	13	241	18.54	19	Wang, Y	113
10	Feng, Biao	Western University	12	974	81.17	22	Joussen, Am	110

**Table 4:** Top 10 most productive journals.

**Rank**	**Journals**	**Documents**	**Total Citations**	**Average Citations**	**IF (2023)**	**JCR**
1	Investigative Ophthalmology & Visual Science	57	3088	54.18	5	Q1
2	Experimental Eye Research	37	980	26.49	3	Q1
3	Scientific Reports	25	539	21.56	3.8	Q1
4	Journal of Cellular Physiology	22	847	38.50	4.5	Q1
5	Molecular Vision	22	698	31.73	1.8	Q4
6	Frontiers in Endocrinology	20	337	16.85	3.9	Q2
7	Diabetes	19	1817	95.63	6.2	Q1
8	Current Eye Research	18	412	22.89	1.7	Q3
9	International Journal of Molecular Sciences	18	238	13.22	4.9	Q1
10	Biochemical And Biophysical Research Communications	16	457	28.56	2.5	Q3

**Table 5:** Top 10 co-citation references.

**Rank**	**Titles**	**First Author**	**Journals**	**Year of Publication**	**Citations**
1	lncRNA H19 prevents endothelial-mesenchymal transition in diabetic retinopathy	Anu A Thomas	Diabetologia	2019	55
2	Roles of miRNAs and long noncoding RNAs in the progression of diabetic retinopathy	Qiaoyun Gong	Bioscience Reports	2017	50
3	Pathogenic role of microRNA-21 in diabetic retinopathy through downregulation of PPARα	Qian Chen	Diabetes	2017	48
4	MicroRNAs as biomarkers of diabetic retinopathy and disease progression	Bridget Martinez	Neural Regeneration Research	2019	46
5	Diabetic Retinopathy: Pathophysiology and Treatments	Wei Wang	International Journal of Molecular Sciences	2018	45
6	Downregulation of circRNA DMNT3B contributes to diabetic retinal vascular dysfunction through targeting miR-20b-5p and BAMBI	Ke Zhu	EBioMedicine	2019	40
7	Role of Inflammation in Diabetic Retinopathy	Anne Rübsam	International Journal of Molecular Sciences	2018	39
8	MALAT1: An Epigenetic Regulator of Inflammation in Diabetic Retinopathy	Saumik Biswas	Scientific Reports	2018	38
9	Role of microRNAs in the modulation of diabetic retinopathy	Rodolfo Mastropasqua	Progress in Retinal and Eye Research	2014	37
10	lncRNA-MIAT regulates microvascular dysfunction by functioning as a competing endogenous RNA	Biao Yan	Circulation Research	2015	37

**Table 6:** RNA in the top 10 most cited research articles.

**RNA Type**	**Role of RNA in DR**	**Mechanisms**	**DOI**
lncRNA-H19	lncRNA-H19 prevents endothelial-mesenchymal transition in diabetic retinopathy.	H19 overexpression prevents glucose-induced endothelial-mesenchymal transition through TGF-β1 and subsequent TGF-β signalling *via* a blockade of the MAPK–ERK1/2 pathway.	10.1007/s00125-018-4797-6
microRNA-21	Knockout of miR-21 alleviates microvascular damage, ameliorates inflammation, and reduces cell apoptosis in the retina of db/db mice.	Knockout of miR-21 prevents the decrease of peroxisome proliferator–activated receptor-α.	10.2337/db16-1246
circRNA-DMNT3B	circDNMT3B overexpression reduces retinal acellular capillary number in diabetic rats.	Downregulation of circDNMT3B contributes to retinal vascular dysfunction in diabetic retinas through regulating miR-20b-5p and BAMBI.	10.1016/j.ebiom.2019.10.004
lncRNAs-MALAT1	MALAT1 can influence the expression of retinal inflammatory transcripts in diabetic patients	MALAT1 influences the expression of inflammatory transcripts through its association with epigenetic mediators, such as histones and DNMT.	10.1038/s41598-018-24907-w
lncRNA-MIAT	lncRNA-MIAT knockdown ameliorates diabetes mellitus–induced retinal microvascular dysfunction *in vivo*, and inhibites endothelial cell proliferation, migration, and tube formation *in vitro*.	lncRNA-MIAT functioned as a ceRNA, which may sequester miR-150-5p, thereby relieving its repressive effect on VEGF expression.	10.1161/CIRCRESAHA.116.305510

**Table 7:** Top 10 keywords ranked by frequency.

**Rank**	**Keywords**	**Count**	**Rank**	**Keywords**	**Count**
1	Diabetic retinopathy	704	6	Micrornas	111
2	Endothelial growth factor	138	7	Vegf	105
3	Angiogenesis	122	8	Endothelial cells	103
4	Activation	112	9	Retinopathy	93
5	Apoptosis	111	10	Mechanisms	85

## Data Availability

The authors confirm that the data supporting the findings of this study are available within the article.

## LIST OF ABBREVIATIONS

BAMBI: BMP and Activin Membrane-Bound Inhibitor
DR: Diabetic Retinopathy
EVs: Extracellular Vesicles
HRMECs: Human Retinal Microvascular Endothelial Cells
JCR: Journal Citation Reports
ncRNAs: Non-Coding RNAs
RMECs: Retinal Microvascular Endothelial Cells
VEGF: Vascular Endothelial Growth Factor
WoSCC: Web of Science Core Collection
ZO-1: Zonula Occludens 1

## STANDARDS OF REPORTING

PRISMA guidelines were followed.

## SUPPLEMENTARY MATERIAL

PRISMA checklist is available as supplementary material on the publisher’s website along with the published article.
